# Utilization of a biosorbent derived from plant residues for the treatment of water contaminated with rhodamine B: preparation and characterization

**DOI:** 10.1039/d5ra07130e

**Published:** 2025-11-06

**Authors:** Ahlem Sarra Saadi, Djihane Slimane Ben Ali, Salim Bousba

**Affiliations:** a Department of Process Engineering, Faculty of Technology, University 20-August-1955 Skikda Algeria as.saadi@univ-skikda.dz sara.ahlem21@gmail.com; b LRPCSI- Laboratoire de Recherche sur la Physico-Chimie des Surfaces et Interfaces, Université du 20 Août 1955 BP. 26, Route d’El-Hadaiek Skikda Algeria; c Département de Génie des Procédés, Faculté de Génie des Procédés, Université Salah Boubnider Constantine 3 Algeria; d Laboratoire Médicament et Développement Durable ReMeDD, Université Salah Boubnider Constantine 3 Algeria

## Abstract

This study reports the preparation and characterization of a novel biosorbent obtained from oak pericarp for the removal of rhodamine B (RhB) dye from aqueous solutions. Comprehensive analyses (FTIR, SEM-EDX, TGA, BET, XRD, and pH_pzc_) confirmed the presence of diverse functional groups and a heterogeneous surface morphology contributing to adsorption. Batch experiments were performed under varying operational conditions (pH, contact time, adsorbent dosage, ionic strength, temperature, and initial concentration), with optimal adsorption achieved at pH 2, using 0.05 g L^−1^ of biosorbent and an equilibrium time of 300 min. Adsorption equilibrium data were best fitted by the Langmuir–Freundlich isotherm, suggesting heterogeneous and multilayer adsorption, with a maximum capacity of 160.809 mg g^−1^ at 20 °C. Kinetic modeling indicated that the pseudo-second-order model best described the process, pointing to chemisorption as the dominant mechanism. Thermodynamic results confirmed the spontaneous and endothermic nature of RhB adsorption. Furthermore, regeneration tests revealed that the biosorbent retained high efficiency over multiple cycles, demonstrating its potential as a sustainable, low-cost, and environmentally friendly material for wastewater treatment.

## Abstract 2

An eco-friendly biosorbent made from oak shells was developed and used to remove rhodamine B from water, showing the promise of natural waste materials for sustainable wastewater treatment.

## Graphical abstract

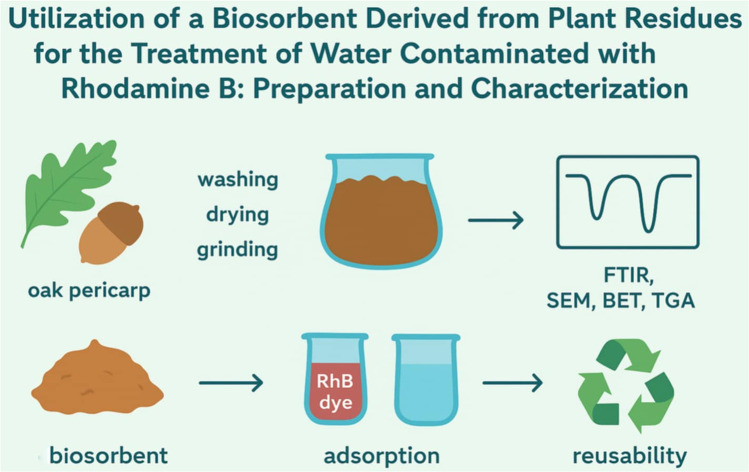

## 1. Introduction

Water pollution remains one of the most pressing global environmental challenges, mainly due to the discharge of untreated industrial effluents into aquatic ecosystems. Industrial effluents from textiles, leather, cosmetics, and food processing industries are often rich in synthetic dyes, which are persistent, non-biodegradable, and potentially toxic.^1,2^

Among these pollutants, rhodamine B (RhB) stands out as a cationic xanthene dye known for its stability, vivid color, and environmental persistence. Its presence in water bodies not only reduces photosynthetic activity by limiting light penetration but also poses health hazards, as it is potentially toxic, carcinogenic, and mutagenic.^3,4^ Dyes are generally classified into categories such as reactive, direct, basic, disperse, acid, vat, and azo dyes, each exhibiting distinct chemical structures and environmental impacts.^5^

Conventional water treatment methods—including coagulation–flocculation, ion exchange, advanced oxidation processes, photocatalysis, sonochemical treatments, membrane separation, and reverse osmosis—have been widely applied. However, they often suffer from high operational costs, incomplete removal, or secondary pollution issues.^6,7^ In contrast, adsorption, particularly using biosorbents derived from agricultural or plant waste, has emerged as an attractive, environmentally friendly, and cost-effective alternative. Such biosorbents typically exhibit abundant functional groups (hydroxyl, carboxyl) and porous structures that facilitate the effective removal of organic dyes.^8,9^

Recently, attention has shifted toward sustainable, plant-based biosorbents, which present a green and nature-based solution for dye removal. These biomass-derived adsorbents offer several advantages, including low cost, abundance, ease of regeneration, high affinity for diverse pollutants, and minimal risk of secondary contamination.^10,11^

Nonetheless, one of the main limitations in conventional adsorbents is the difficulty of separating them from treated water. Applications of such biosorbents for RhB removal have been demonstrated using a variety of plant-derived materials for instance: banana peel powder, which displayed removal efficiencies up to 81% and followed Langmuir isotherm behavior;^12^ macroalgal biomass, modified or native, achieving capacities over 100 mg g^−1^ under acidic conditions;^13^ rice husk biochar, reaching removal efficiencies near 98% under optimized conditions, with monolayer physisorption behavior and feasible regeneration.^14^

These findings underscore the potential of plant-waste-based biosorbents, but they also highlight the need for new materials with optimized properties tailored for RhB adsorption, particularly in terms of adsorption capacity, selectivity, and reusability.

Oak acorn pericarp, an abundant lignocellulosic by-product in Algeria, has not yet been extensively explored as a biosorbent. Its heterogeneous surface structure and rich functional groups make it a promising candidate for dye removal. In this study, a biosorbent derived from oak acorn pericarp was prepared through a simple process and characterized using FTIR, SEM-EDX, BET, TGA, XRD, and pHpzc analyses to elucidate its physicochemical properties. The adsorption performance toward rhodamine B was evaluated under varying conditions of pH, contact time, adsorbent dosage, and temperature. Kinetic, isotherm, and thermodynamic analyses were conducted to clarify the adsorption mechanism, while reusability tests assessed its potential for practical wastewater treatment applications.

## 2. Experimental

### 2.1. Materials

The primary raw material utilized in this study was the pericarp of oak acorns (*Quercus*). The chemical reagents included zinc chloride (ZnCl_2_, BIOCHEM Chermopharma), sodium hydroxide (NaOH, SPECILAB), hydrochloric acid (HCl, Honeywell Fluka), rhodamine B dye (C_28_H_31_ClN_2_O_3_, BIOCHEM Chermopharma), sodium chloride (NaCl, BIOCHEM Chermopharma), and calcium chloride (CaCl_2_, BIOCHEM Chermopharma).

The concentration of rhodamine B before and after contact with the oak acorn pericarp was measured using a UV-visible spectrophotometer (SHIMADZU UV-1900i). All aqueous solutions were prepared using deionized (DI) water.

### 2.2. Preparation of a biosorbent from oak acorn pericarp

The pericarp of oak acorns was collected from the village of Ain Lamsid, located in the municipality of Wadi Al-Zuhur, in the westernmost part of Skikda Province. The collected material was rinsed several times with clean water, followed by distilled water to remove any remaining impurities.

After washing, the pericarp was dried in an oven at 378 K for 24 hours. The dried biomass was then ground and sieved to obtain particles with sizes of ≤500 μm, ≤180 μm, and ≤63 μm.

### 2.3. Biosorbent characterization

The physicochemical properties of the oak acorn pericarp biosorbent were investigated using a combination of advanced analytical techniques, including Fourier Transform Infrared Spectroscopy (FTIR), Scanning Electron Microscopy coupled with Energy Dispersive X-ray Spectroscopy (SEM-EDX), Brunauer–Emmett–Teller (BET) surface area analysis, and Thermogravimetric Analysis (TGA).

FTIR analysis (Bruker) was performed to identify the functional groups present on the surface of the biosorbent both before and after the adsorption of rhodamine B dye. The infrared spectra were recorded in the range of 400 to 4000 cm^−1^, allowing for the detection of characteristic vibrational bands associated with key functional moieties involved in the adsorption mechanism.

SEM-EDX analysis (Quattro S) was employed to examine the surface morphology and elemental composition of the biosorbent. Micrographs were taken before and after the biosorption process to evaluate surface texture, porosity, and any structural changes, while the EDX detector provided qualitative and semi-quantitative information on the elemental distribution on the biosorbent surface.

Brunauer–Emmett–Teller (BET) surface area analysis (Quantachrome Autosorb-iQ3) was conducted to determine the specific surface area, pore volume, and pore size distribution of the biosorbent. This analysis was critical for assessing the porous nature and the physical adsorption potential of the material.

Thermogravimetric analysis (TGA, METTLER TOLEDO) was carried out to evaluate the thermal stability and decomposition behavior of the biosorbent. The analysis was performed under a nitrogen atmosphere, with a heating rate of 10 °C min^−1^ over a temperature range of 20 to 1000 °C. The TGA profile provided insights into moisture content, volatile matter release, and the thermal degradation profile of the biomass.

### 2.4. Determination of the point of zero charge (pH_pzc_)

The point of zero charge (pH_pzc_) of the biosorbent was determined using the batch equilibrium method. The ionic strength of the solution was maintained constant by using a 0.1 M Sodium chloride (NaCl) solution.

A series of 50 mL NaCl solutions with initial pH values ranging from 2 to 12 were prepared by adjusting the pH with either 0.1 M Hydrochloric acid (HCl) or 0.1 M Sodium hydroxide (NaOH). Subsequently, 0.1 g of the oak acorn pericarp biosorbent was added to each solution. The suspensions were stirred continuously at 250 rpm and kept at ambient temperature (approximately 25 °C) for 48 hours to reach equilibrium.

After equilibration, the oak acorn pericarp biosorbent was removed by filtration or decantation, and the final pH of each solution was recorded. The difference between the final and initial pH values (ΔpH = pH_final_ − pH_initial_) was plotted as a function of the initial pH. The pH at which ΔpH equals zero corresponds to the point of zero charge (pH_pzc_) of the biosorbent.^15^

### 2.5. Batch biosorption studies

Batch adsorption experiments were performed to evaluate the biosorption performance of the oak acorn pericarp for rhodamine B (RhB) dye under various operating conditions. The effects of several parameters were investigated, including initial solution pH, contact time, biosorbent dose, temperature, initial dye concentration, and ionic strength (salt effect). All experiments were conducted at a constant temperature of 20 °C.

For each experiment, a fixed amount of oak acorn pericarp biosorbent (50 mg) with a particle size of ≤63 μm was added to 100 mL Erlenmeyer flasks containing 50 mL of RhB dye solution at an initial concentration of 120 mg L^−1^. The flasks were sealed with aluminum foil to prevent contamination or evaporation and placed on a magnetic stirrer (DIAB MS-H-S) operating at 300 rpm. Agitation was continued until equilibrium was reached.

The influence of initial solution pH on dye adsorption was studied over a pH range of 2 to 12. pH adjustments were made using 0.1 M HCl or 0.1 M NaOH as required. The contact time between the biosorbent and the dye solution was varied from 5 to 300 minutes to examine adsorption kinetics. Additionally, the effect of the initial dye concentration was evaluated by preparing RhB solutions in the range of 10 to 1000 mg L^−1^. The influence of salt ionic strength on the biosorption capacity of the oak acorn pericarp was examined by adding different concentrations of sodium chloride (NaCl) and potassium chloride (KCl) to the rhodamine B (RhB) dye solution.

The effect of temperature on RhB adsorption was evaluated by conducting a series of batch experiments at an initial dye concentration of 120 mg L^−1^ over a temperature range of 20 to 60 °C. These experiments were designed to assess the thermal dependency of the adsorption process and to enable thermodynamic analysis.

**Fig. 1:**
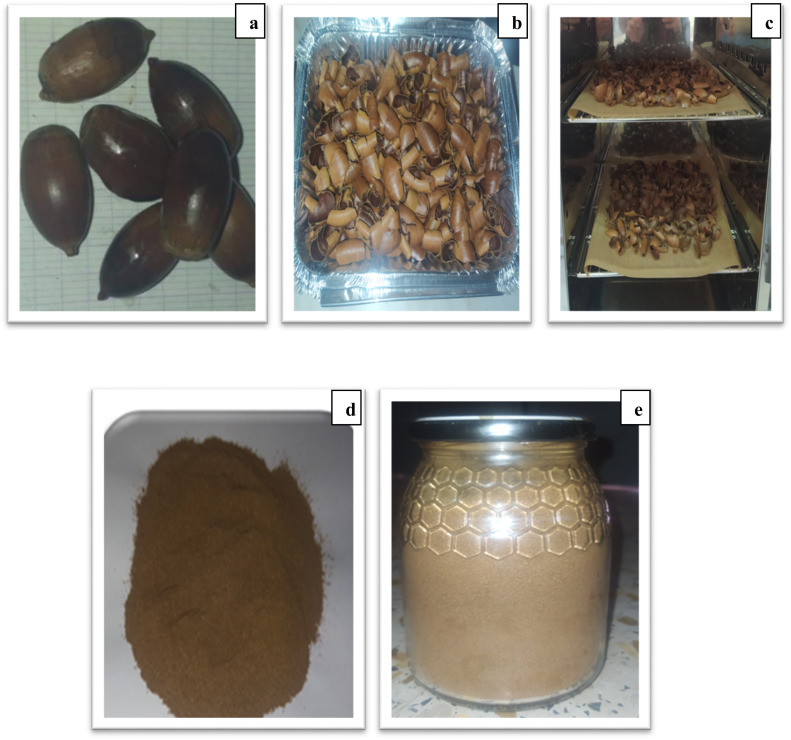
Schematic representation of the preparation steps of a biosorbent from oak acorn pericarp, including collection (a), washing (b), drying (c), grinding (d), and sieving into different particle sizes (e).

All adsorption experiments were carried out in triplicate under identical conditions to ensure reproducibility. The mean values are presented with corresponding standard deviation error bars in all figures, confirming the statistical reliability of the obtained data (Fig. 1).

### 2.6. Adsorption isotherms, kinetics, and thermodynamics

To study adsorption isotherms, additional experiments were performed using a range of initial RhB concentrations from 10 to 500 mg L^−1^ under optimal conditions. The adsorption kinetics were investigated by monitoring the amount of dye adsorbed at various time intervals, ranging from 5 to 300 minutes, in order to model the rate and mechanism of the process.

Following each adsorption test, the mixture was centrifuged at 4000 rpm for 15 minutes using a centrifuge (ONiLAB DM0412). The supernatant was carefully decanted and analyzed to determine the residual dye concentration using a double-beam UV-visible spectrophotometer (SHIMADZU UV-1900i) at the maximum absorbance wavelength of RhB (554 nm).

The amount of dye adsorbed at equilibrium (*q*_e_, in mg g^−1^) and the percentage removal efficiency (*R*%) were calculated using the following equations:1
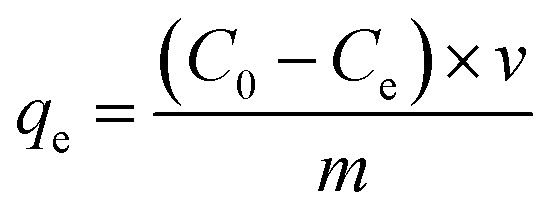
2
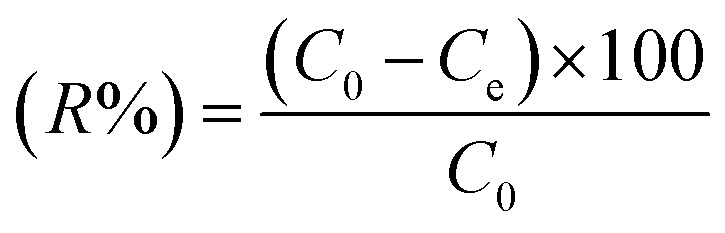
where: *C*_0_ and *C*_e_ are the initial and equilibrium concentrations of RhB (mg L^−1^), *V* is the volume of the solution (L), *m* is the mass of the biosorbent (g).

Adsorption equilibrium data were fitted to several isotherm models, including Langmuir, Freundlich, Temkin, and Langmuir–Freundlich. Kinetic studies were analyzed using pseudo-first-order and pseudo-second-order models. Thermodynamic parameters were evaluated using the Van't Hoff equation to determine changes in Gibbs free energy (Δ*G*°), enthalpy (Δ*H*°), and entropy (Δ*S*°) associated with the adsorption process.

### 2.7. Reusability experiments

The regeneration and reuse of biosorbents are crucial for their practical application in wastewater treatment. Various regenerating agents, including acids, bases, and organic solvents (ethanol, methanol, acetone), have been employed to desorb retained dyes.^16,17^

In this study, ethanol was used as an efficient regenerant, where the dye-loaded biosorbent was dried, immersed in ethanol under continuous stirring to promote desorption, rinsed with distilled water to remove residual dye and solvent, and subsequently dried for reuse. This regeneration cycle was repeated to ensure stability and reusability. Ethanol-based regeneration proved effective in restoring the adsorption performance of the biosorbent, thereby extending its service life and offering a cost-effective and sustainable approach for dye removal from contaminated water.^18^

## 3. Results and discussion

### 3.1. Characterization of the biosorbent

#### 3.1.1. Analysis of functional groups (FTIR)

**Fig. 2:**
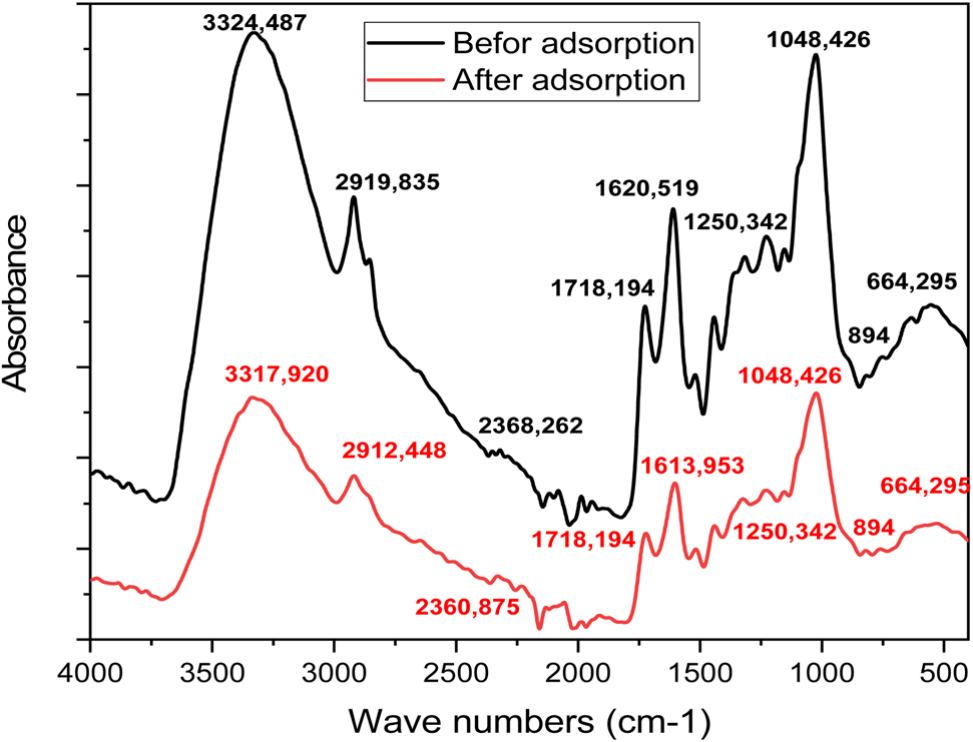
FTIR spectra of the oak pericarp biosorbent before and after RhB dye adsorption.

The FTIR spectra of oak acorn pericarp samples before and after the adsorption of rhodamine B (RhB) dye are shown in Fig. 2. The spectra reveal characteristic bands of aromatic and aliphatic functional groups typical of lignocellulosic materials, mainly originating from cellulose, hemicellulose, and lignin. The biosorbent exhibits various oxygenated functional groups, such as olefins, esters, ketones, aromatic rings, and alcohols. Notable absorption bands were recorded at 3400–3200 cm^−1^ (O–H stretching), 1767–1718 cm^−1^ (C=O stretching), 1250 cm^−1^ (C–O–C stretching), and 1048 cm^−1^ (C–O–H bending).^19^ A band at 1620 cm^−1^ corresponds to C=C stretching in aromatic compounds, while the absorption at 2919 cm^−1^ is assigned to symmetric C–H stretching and CH_2_ vibrations. The peak at 894 cm^−1^ is associated with C–H deformation in cellulose, a typical feature of the lignocellulosic matrix^20^

After RhB adsorption, several peaks exhibited slight shifts, including 1620 to 1613 cm^−1^, 2368 to 2360 cm^−1^, 2919 to 2912 cm^−1^, and 3324 to 3318 cm^−1^. Additionally, a decrease in the intensity of the –OH and C=O bands was observed, indicating their active participation in RhB binding. These spectral changes confirm the involvement of O–H, C=O, and C–H functional groups in the adsorption process and highlight their role in the interaction between RhB dye molecules and the oak acorn pericarp biosorbent.

#### 3.1.2. Biosorbent morphology and porous properties (SEM/EDX)

**Fig. 3:**
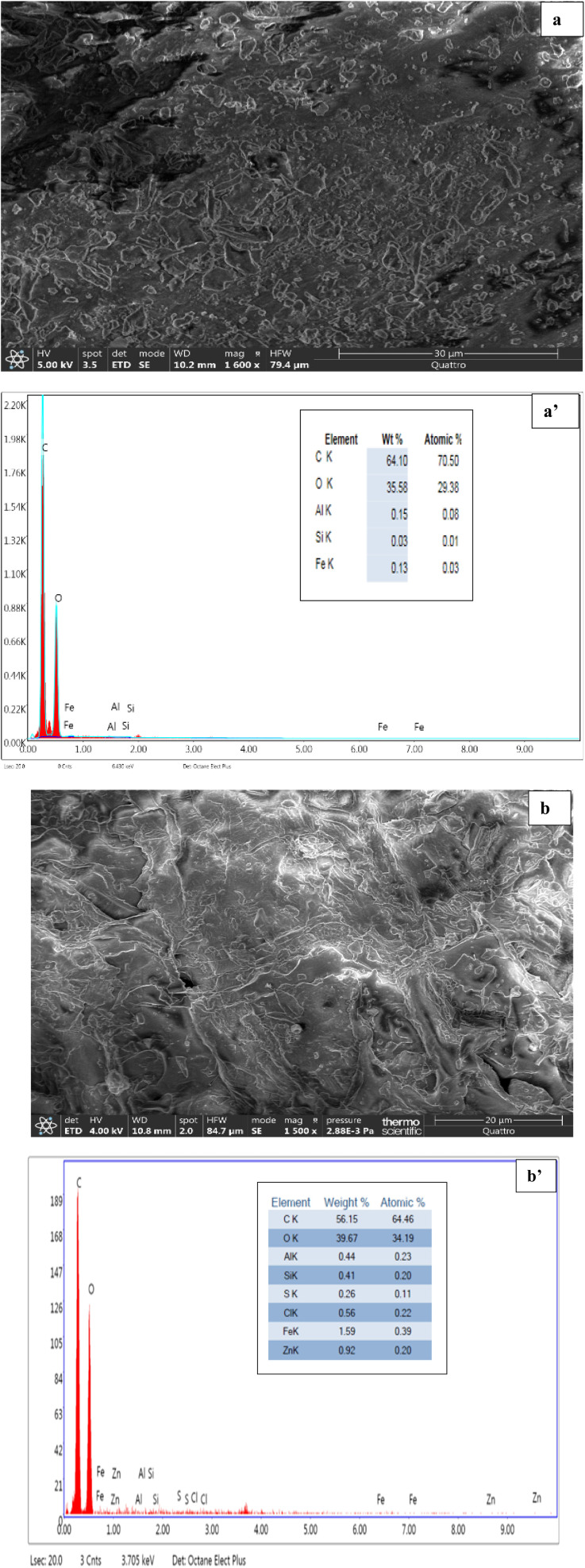
SEM and EDX of oak pericarp biosorbent before (a and a′) and after (b and b′) RhB dye adsorption.

Scanning electron microscopy (SEM) and energy-dispersive X-ray spectroscopy (EDX) analyses were employed to investigate the morphological features and surface characteristics of the oak pericarp before and after RhB dye adsorption. Fig. 3(a), captured at 1600× magnification, reveals that the raw oak pericarp exhibits a highly porous structure with small particle size. The surface morphology is rough, irregular, and heterogeneous, with abundant pores, which can enhance the number of active adsorption sites.^21^ Such characteristics are advantageous for biosorption, as they promote multiple interactions between the biosorbent surface and pollutant molecules.

After contact with RhB dye, notable morphological changes were observed, as shown in Fig. 3(b) (1500× magnification).^22^ The biosorbent surface became less rough and exhibited reduced heterogeneity, suggesting that the dye molecules occupied and partially blocked the pores.

EDX analysis further revealed significant changes in the elemental composition of the oak pericarp after RhB adsorption. Prior to adsorption, the biosorbent was predominantly composed of carbon, oxygen, and iron, with smaller amounts of aluminum, silicon, and sulfur. Following adsorption, variations in peak intensities were observed, indicating the attachment of dye molecules and possible ion exchange processes at the biosorbent surface.^23^

The detection of zinc in the EDX spectra after adsorption may result from residual ZnCl_2_ used during the activation step. Despite extensive washing, trace amounts of zinc can remain weakly bound within the carbon matrix, which may explain its appearance in the spectrum.

#### 3.1.3. Thermogravimetric analysis (TGA)

**Fig. 4:**
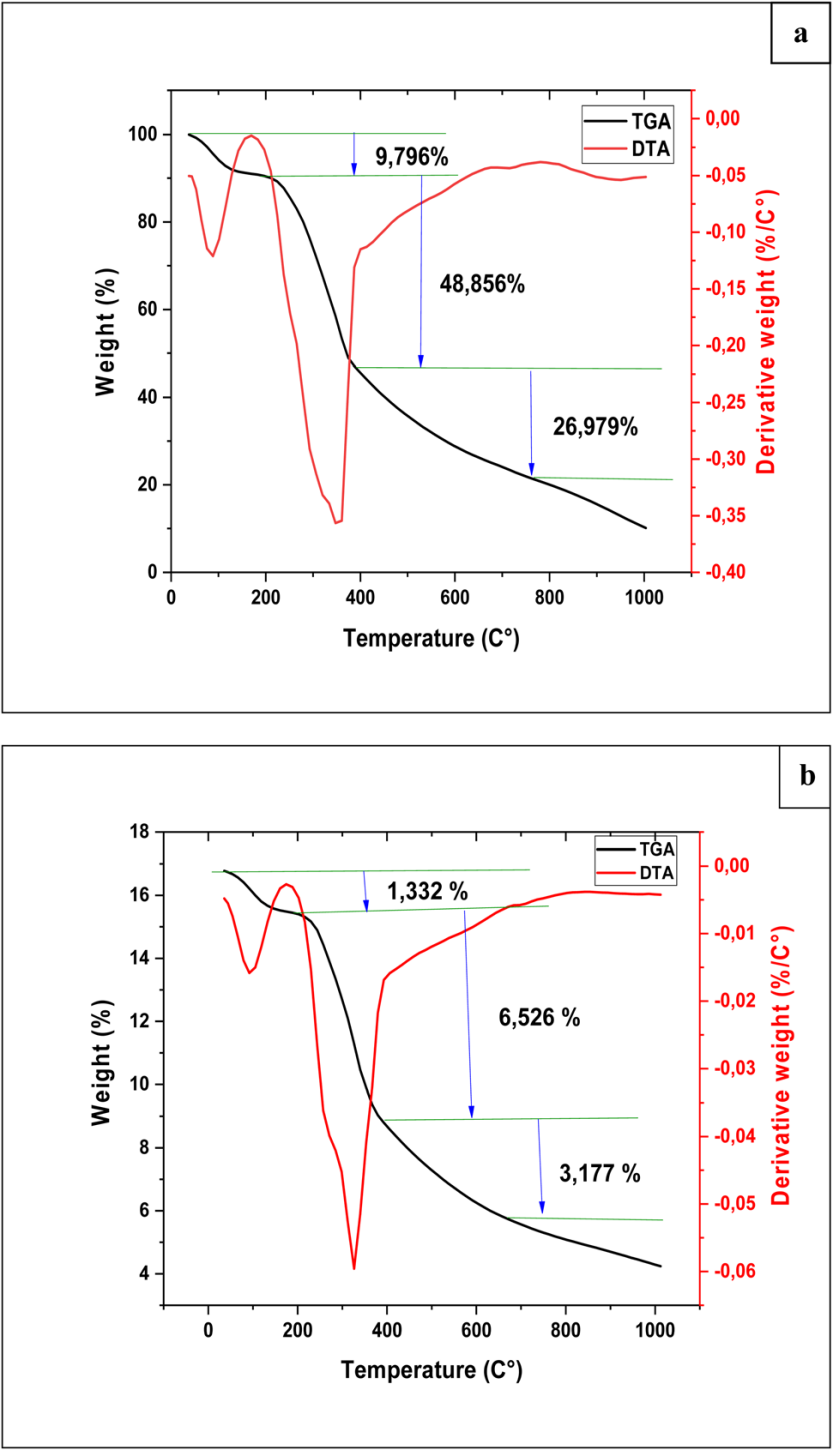
Thermogravimetric analysis of oak pericarp biosorbent before (a) and after (b) RhB dye adsorption.

Thermogravimetric analysis (TGA) is a technique used to monitor mass loss as a function of temperature.^24^ The TGA curve of the oak pericarp, presented in Fig. 4, exhibits three main stages of thermal degradation.The first stage occurs below 200 °C, showing a mass loss of approximately 9.80%, which is attributed to the evaporation of moisture physically adsorbed on the surface and within the pores of the biosorbent.The second stage begins above 200 °C and reaches a maximum decomposition rate at 378.12 °C, corresponding to a mass loss of 48.86%. This stage mainly involves the degradation of hemicellulose (200–300 °C), followed by cellulose decomposition (300–400 °C). Between 250 °C and 400 °C, a total weight loss of 52.57% is observed, which is associated with the partial degradation of water-soluble organic compounds.^23^

The third stage, occurring between 400 °C and 1000 °C, is attributed to the decomposition of lignin, resulting in a mass loss of 26.98%.^24^ The decomposition temperatures and relative weight losses are influenced by the intrinsic composition of the material and any chemical treatments applied during preparation.

Furthermore, the high thermal stability observed in the TGA curve supports the amorphous and thermally resistant nature of the biosorbent revealed by XRD analysis, confirming its structural robustness and suitability for adsorption applications under variable environmental conditions.

#### 3.1.4. Brunauer–Emmett–Teller analysis (BET)

**Fig. 5:**
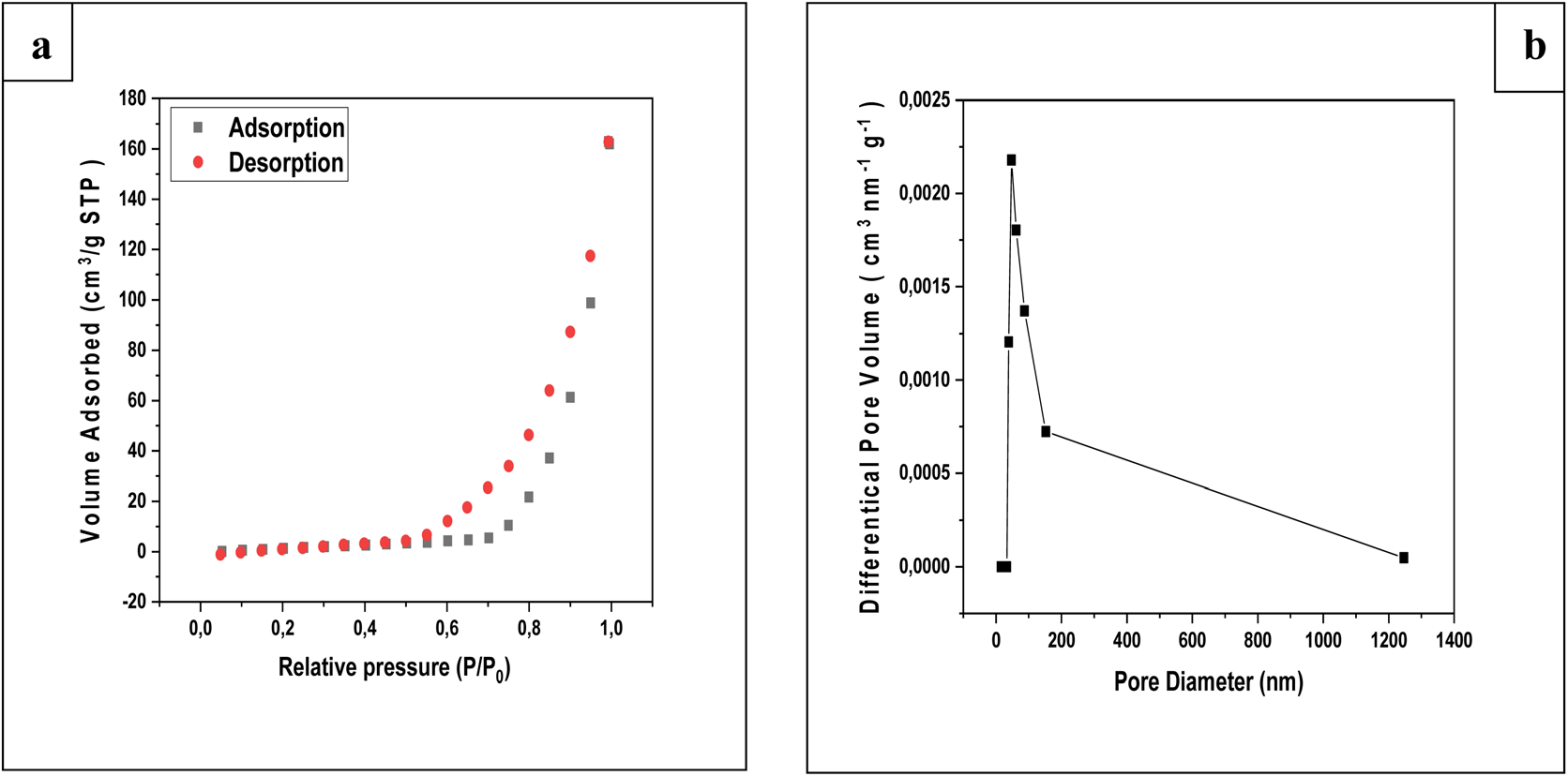
Nitrogen adsorption – desorption isotherms (a), pore volume distributions (b) of the oak pericarp biosorbent.

The Brunauer–Emmett–Teller (BET) method was applied to determine the specific surface area of the oak pericarp biosorbent, while pore size distribution (PSD) was evaluated using the Barrett–Joyner–Halenda (BJH) method. Fig. 5(a) presents the N_2_ adsorption–desorption isotherms, and Fig. 5(b) shows the corresponding pore size distribution.

The isotherm curve displays a distinct H1-type hysteresis loop, indicating the presence of mesoporous structures within the biosorbent.^25^ Such pore geometry is advantageous for adsorption processes, as it provides accessible channels for pollutant molecules. The BJH analysis, based on the desorption branch of the nitrogen isotherm, confirmed the mesoporous nature of the oak pericarp.^25^

Before RhB dye adsorption, the oak pericarp exhibited a specific surface area of 45.416 m^2^ g^−1^. After adsorption, the surface area increased markedly to 319.003 m^2^ g^−1^. This substantial increase suggests that the adsorption of RhB involves electrostatic interactions and the activation of new surface sites, enhancing the biosorbent's capacity to remove dye molecules from aqueous solutions.^22^

#### 3.1.5. X-ray diffraction (XRD)

**Fig. 6:**
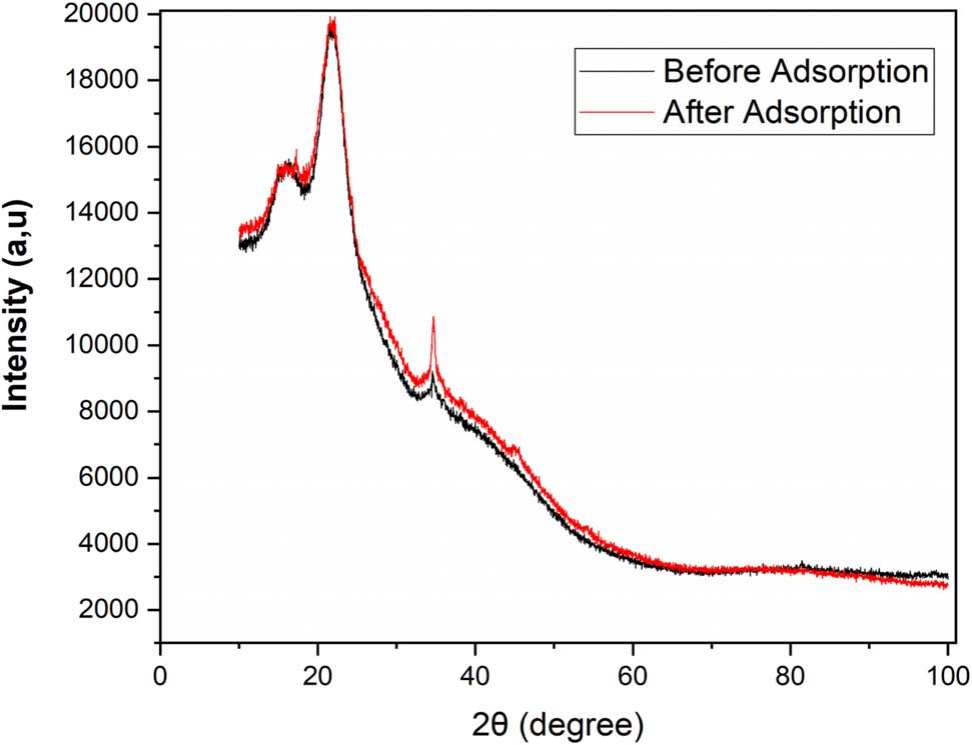
X-ray diffraction patterns of the oak pericarp biosorbent before and after dye adsorption.

The XRD patterns of the biosorbent before and after adsorption are shown in Fig. 6. Both diffraactograms exhibit a broad diffraction hump centered around 2*θ* ≈ 20–30°, which is characteristic of amorphous carbonaceous materials.^26^ The absence of sharp crystalline peaks confirms the predominantly amorphous nature of the prepared adsorbent.

After adsorption, no significant shift in the diffraction peak positions was observed, indicating that the overall crystalline framework of the biosorbent remained intact. However, a slight decrease in peak intensity can be noticed, which may be attributed to the surface coverage of the biosorbent by the adsorbed metal ions and possible partial filling of the porous sites.^27^ This behavior suggests that the adsorption process mainly occurs on the surface functional groups and pores of the material without altering its bulk crystallinity.

The XRD results are consistent with the FTIR and BET analyses, confirming that the amorphous structure corresponds to a disordered carbon framework enriched with surface functional groups. These findings are consistent with previous studies reporting that heavy metal ion adsorption onto amorphous carbon-based or polymer-modified composites does not cause major structural transformations but rather induces minor intensity variations due to surface interactions.^28,29^

### 3.2. Point of zero charge (pH_PZC_)

**Fig. 7:**
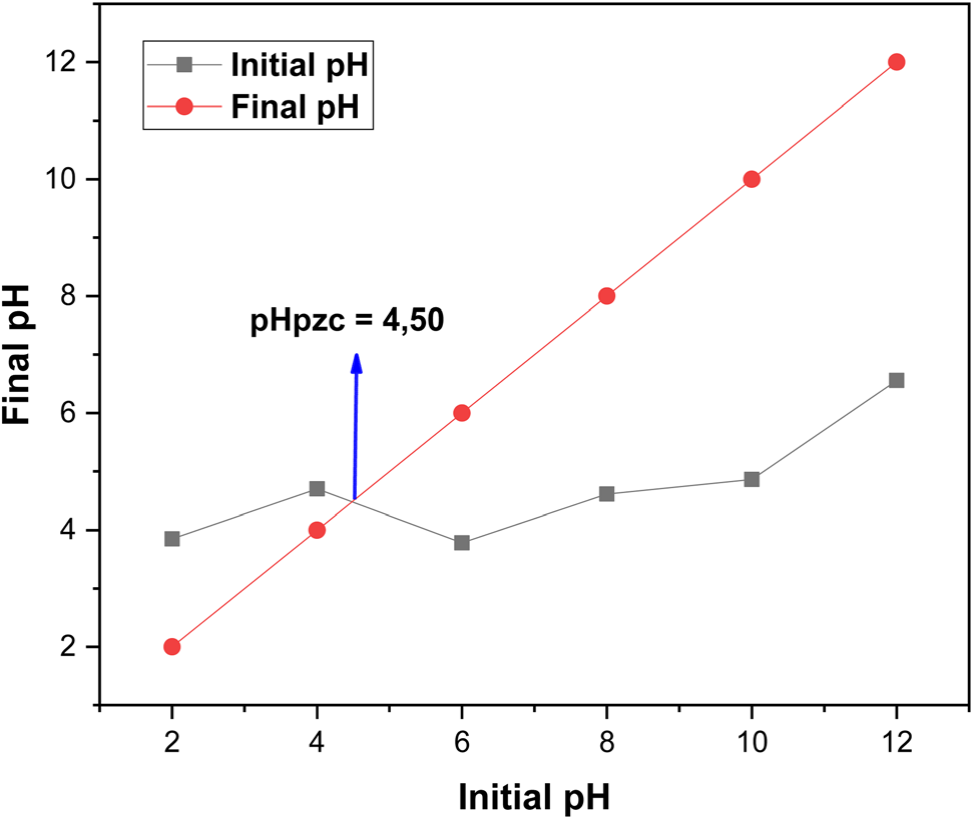
Point of zero charge of oak pericarp biosorbent.

The point of zero charge (pH_pzc_) is a critical parameter in adsorption studies, as it represents the pH at which the net surface charge of the adsorbent is zero (Fig. 7).^30^ For the biosorbent used in this study, the pH_pzc_ was determined to be 4.50.

At pH values below 4.50, the adsorbent surface carries a positive charge, which leads to electrostatic repulsion between cationic species and the positively charged functional groups, thereby reducing adsorption efficiency. In contrast, at pH values above 4.50, the surface becomes negatively charged, creating favorable electrostatic conditions for the adsorption of cationic species.

Therefore, maintaining the solution pH above the pH_pzc_ promotes a negatively charged adsorbent surface, enhancing electrostatic attraction and maximizing cation removal efficiency.^31^

In this study, adsorption experiments were conducted at pH 2, which is below the pH_pzc_ value. Under these conditions, the surface of the biosorbent becomes positively charged, favoring the electrostatic attraction of the negatively charged rhodamine B dye molecules. This explains the high adsorption efficiency observed at low pH values.

Furthermore, understanding the pH_pzc_ provides valuable insights for optimizing the adsorption process at a larger scale, allowing for better control of pH conditions to maximize pollutant removal efficiency and ensure consistent performance in industrial applications.

### 3.3. Batch adsorption experiment

#### 3.3.1. Effect of solution pH

The pH of the solution is a critical parameter affecting the biosorption mechanism, as it influences the surface charge of the biosorbent, the ionization degree of the adsorbate, and the dissociation of the functional groups present on the biosorbent surface. In this study, the effect of pH on RhB removal was investigated under batch conditions.^23^

The point of zero charge (pH_pzc_) of the biosorbent was determined to be 4.5. At pH values lower than the pH_pzc_, the biosorbent surface is positively charged, whereas at pH values higher than the pH_pzc_, it becomes negatively charged. RhB has a p*K*_a_ value of 3.7, meaning that below p*K*_a_ it is primarily in molecular (cationic/monomeric) form, while above p*K*_a_ it predominantly exists in ionic or dimeric forms.

**Fig. 8:**
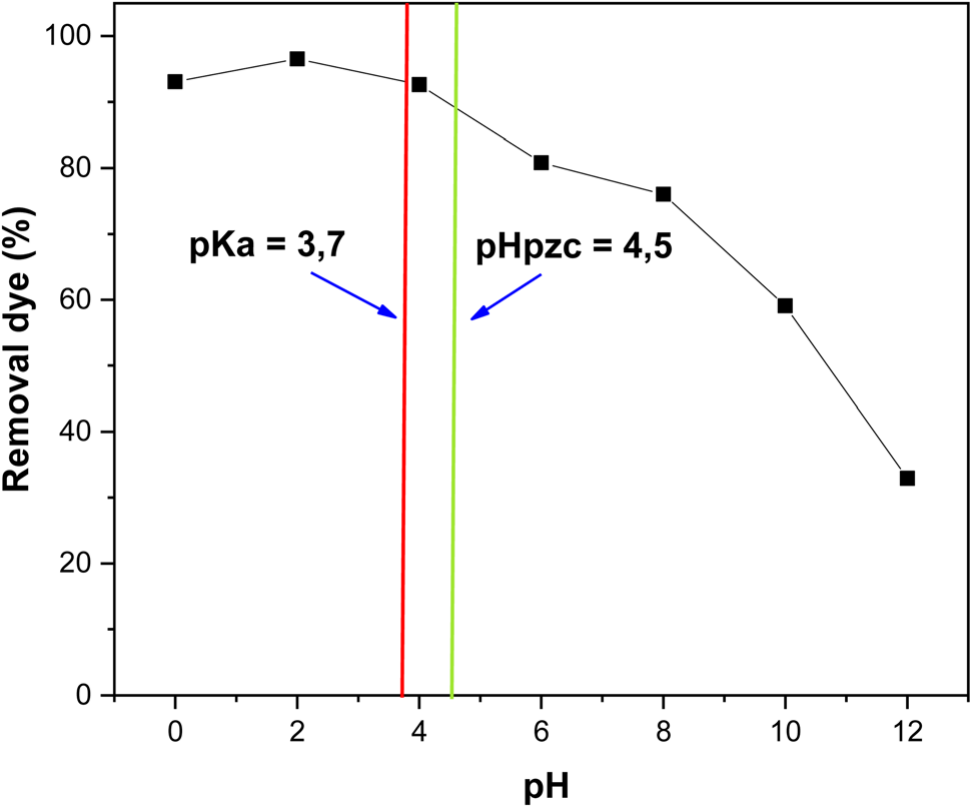
Effect of pH on the removal of RhB dye by oak pericarp biosorbent (*C*_0_ = 120 mg L^−1^; *T* = 20 °C; *t* = 300 min).

^32^Was higher than at other pH values. This suggests that electrostatic interactions were not the sole driving force for biosorption. At low pH, RhB exists mainly in monomeric form, which has a smaller molecular size than the dimeric form, allowing it to diffuse more easily into the micropores of the biosorbent.^30^ Similar observations have been reported by Deshpande & Kumar (2002),^32^ and Gad & El-Sayed (2009).^33^

Experimental results (Fig. 8) show that RhB removal efficiency reached its maximum (*R* ≈ 96.52%) at an initial pH of 2 and gradually decreased with increasing pH, reaching a minimum (*R* ≈ 32.93%) at pH 12. The decrease in removal efficiency at higher pH values can be attributed to the deprotonation of oxygen-containing functional groups (–COOH and –OH) on the biosorbent surface, resulting in negative charges that cause electrostatic repulsion with RhB cations when pH > pH_pzc_.^23^

#### 3.3.2. Effect of biosorbent dose

The biosorbent dose plays a crucial role in adsorption studies, as it determines the number of available active sites and consequently affects the sorption capacity and removal efficiency of the target pollutant.^24^ To determine the optimum dose for rhodamine B (RhB) removal, batch experiments were conducted using biosorbent masses ranging from 0.01 to 0.25 g. Each dose was introduced into 50 mL of RhB solution at room temperature (20 °C) and stirred at 250 rpm under static conditions.^23,24^

**Fig. 9:**
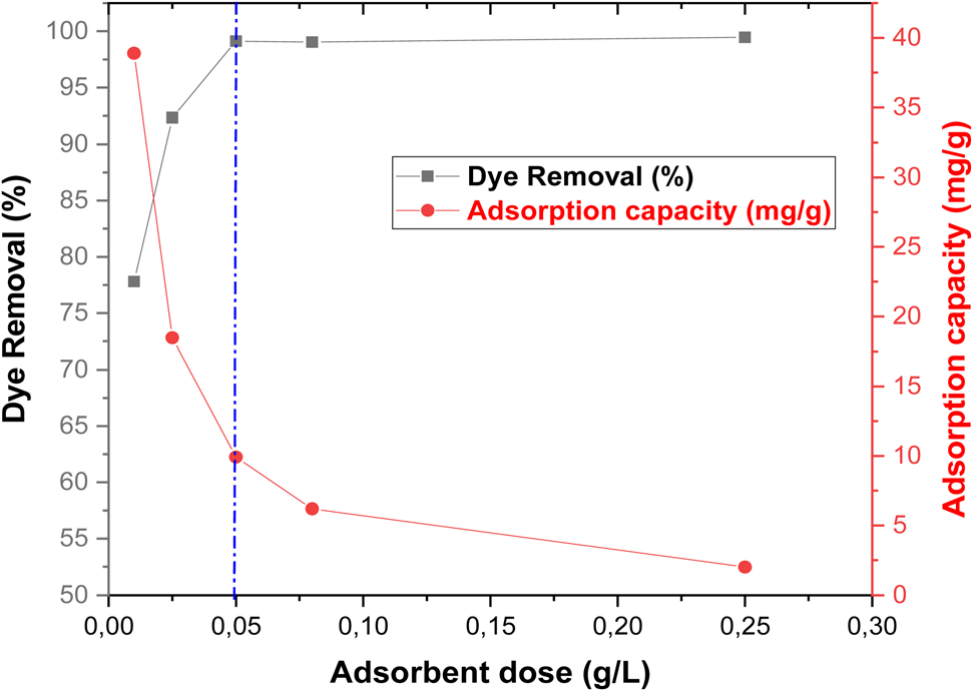
Effect of adsorbent dose on the removal of RhB dye using oak pericarp biosorbent (*C*_0_ = 120 mg L^−1^; *T* = 20 °C; *t* = 300 min).

The results (Fig. 9) show that the dye removal efficiency (*R*%) increased sharply from 77.79% to 99.47% as the biosorbent mass increased from 10 mg to 250 mg. This enhancement can be attributed to the greater number of available active sites, which facilitated increased dye adsorption. However, the adsorption capacity (*q*_e_) exhibited the opposite trend, decreasing with higher biosorbent doses. This inverse relationship is commonly attributed to the aggregation of biosorbent particles at higher dosages, which reduces the available surface area and leads to unsaturated adsorption sites.

Beyond a dose of 50 mg, the increase in removal efficiency became negligible, suggesting that most accessible sites had already been occupied. Therefore, 50 mg was considered the optimum biosorbent dose for RhB removal and was selected for subsequent experiments.^34^

#### 3.3.3. Effect of contact time and initial concentration of RhB dye

**Fig. 10:**
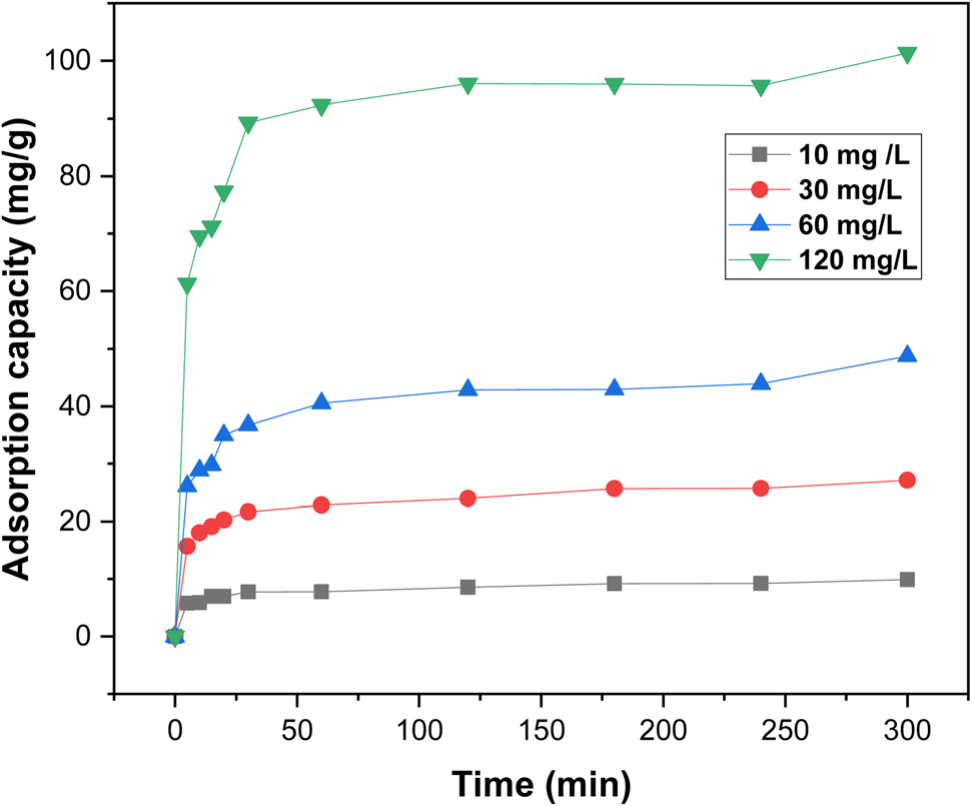
Effect of contact time on the adsorption capacity of RhB dye at various initial concentrations using oak pericarp biosorbent.

Contact time is a critical parameter for the success of any biosorption application. The influence of contact time on RhB dye removal was evaluated at various initial dye concentrations (10–120 mg L^−1^) and contact times ranging from 5 to 300 minutes, as shown in Fig. 10.

The results indicate that adsorption capacity increases with both contact time and initial dye concentration. The removal process started rapidly due to the abundance of available biosorption sites, but progressively slowed down as these sites became saturated.^35^ The equilibrium time was reached at approximately 120 minutes for all tested concentrations. Therefore, the 5 hours contact time used in this study was more than sufficient to ensure equilibrium, and the optimal adsorption period was determined to be around 300 minutes.

Moreover, as the initial RhB concentration increased from 10 to 120 mg L^−1^, the equilibrium adsorption capacity rose from 23.96 to 96 mg g^−1^, indicating the strong dependence of biosorption efficiency on the initial dye concentration.^36^

#### 3.3.4. Effect of salt ionic strength

The ionic strength of the solution is an important parameter in assessing whether hydrophobic–hydrophobic interactions between the non-polar moieties of RhB molecules and those of the adsorbent surface contribute significantly to the adsorption mechanism.^30^

To investigate this effect, adsorption experiments were conducted by varying the concentrations of NaCl and CaCl_2_ from 0.1 to 1.0 mol L^−1^ under otherwise identical conditions.

**Fig. 11:**
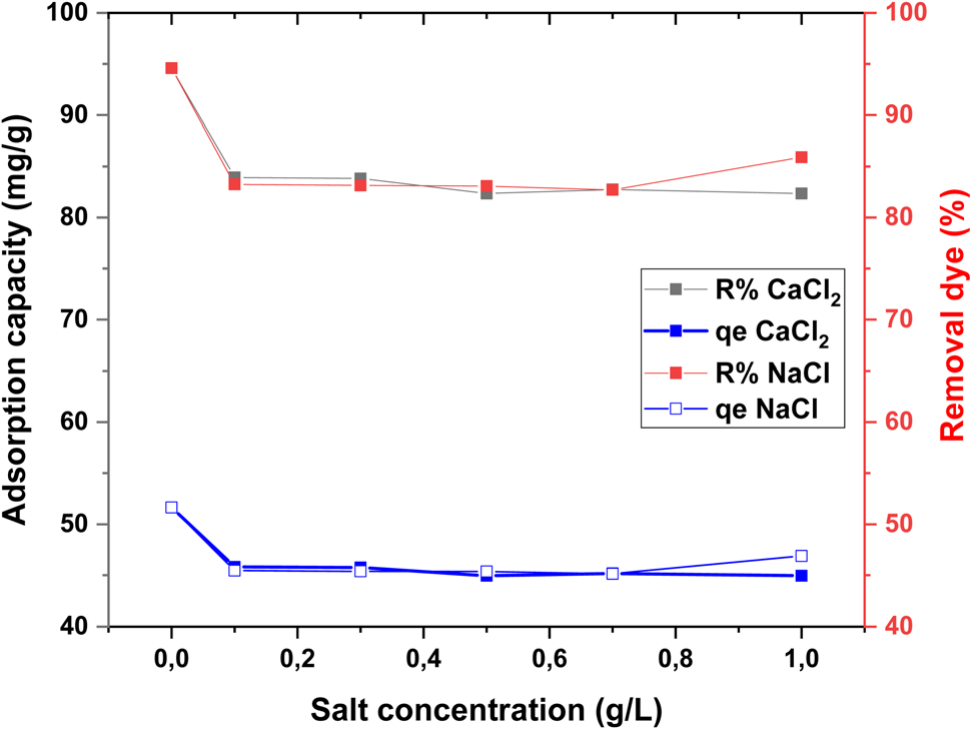
Effect of salt ionic strength on the adsorption of RhB dye (*C*_0_ = 120 mg L^−1^; *T* = 20 °C; *t* = 300 min).

As shown in Fig. 11, the results reveal no significant change in RhB removal efficiency with increasing ionic strength for either salt. This observation suggests that electrostatic interactions are not the dominant driving force in the adsorption process, and that hydrophobic–hydrophobic interactions, if present, are likely weak or masked by other adsorption mechanisms. Similar trends have been reported in previous studies, further supporting the conclusion that ionic strength has a negligible effect on the RhB–adsorbent system under the tested conditions.^36^

#### 3.3.5. Effect of temperature

Temperature is a key parameter influencing the adsorption capacity of biosorbents. The effect of temperature on the removal of RhB by oak pericarp was studied under fixed conditions: initial dye concentration of 120 mg L^−1^, adsorbent dosage of 0.05 g/50 mL, and temperatures ranging from 20 to 60 °C. As shown in Fig. 10, the removal efficiency increased slightly from 89.85% at 20 °C to 91.59% at 60 °C, indicating that the biosorbent maintained high performance across all tested temperatures.^37^

**Fig. 12:**
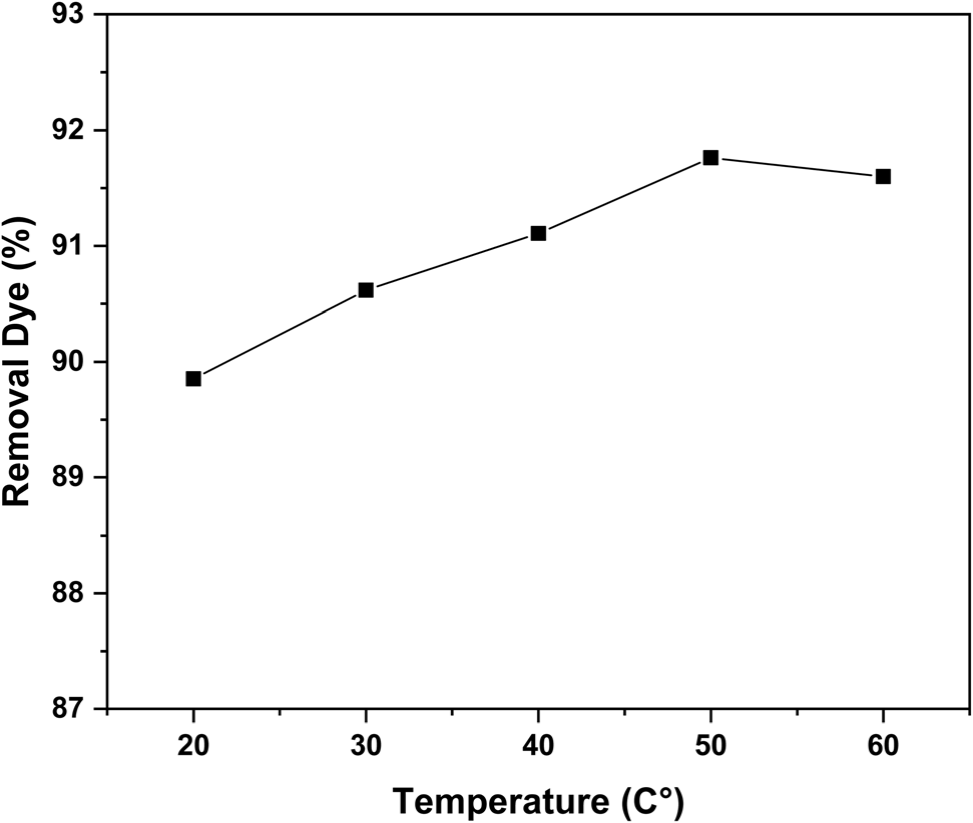
Effect of temperature on the removal efficiency of RhB dye by oak pericarp biosorbent (*m* = 0.05 g, *C*_0_ = 120 mg L^−1^, *t* = 300 min).

The observed enhancement in adsorption efficiency with increasing temperature suggests that the process is endothermic in nature. Higher temperatures may enhance the mobility of RhB molecules and increase the porosity or flexibility of the biosorbent structure, thereby facilitating the diffusion of dye molecules into internal adsorption sites. This thermal activation may also overcome energy barriers associated with the interaction between dye molecules and the active functional groups of the biosorbent (Fig. 12). The high thermal stability of oak pericarp confirms its suitability for dye removal in variable environmental conditions.^19^

### 3.4. Isotherm analysis

Adsorption isotherm models are fundamental for elucidating the nature of interactions between adsorbates and adsorbents, thereby providing key insights into adsorption mechanisms.^24^ Accurate analysis and prediction of adsorption behavior require the correlation of equilibrium data through theoretical or empirical equations. Several isotherm models have been extensively reported in the literature to describe experimental adsorption data.^38^ In the present study, the Langmuir, Freundlich, Temkin and Langmuir–Freundlich models were applied to assess the equilibrium adsorption of RhB dye onto biosorbent at 20 °C, with the determination of regression coefficients (*R*^2^) enabling the identification of the most suitable model for describing the adsorption process.^24^The objective was to evaluate the interaction mechanisms between the dye and the adsorbent, assess the applicability of each model, and determine characteristic parameters for comparing adsorption performance.^38^

The Langmuir isotherm describes monolayer adsorption onto a homogeneous surface with identical binding energies.^39^ Its non-linear form is expressed as follows (Langmuir, 1917):3
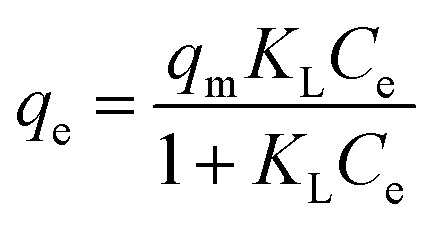
where *C*_e_ (mg L^−1^) is the equilibrium concentration of the adsorbate, *q*_e_ and *q*_m_ (mg g^−1^) are the equilibrium and maximum monolayer adsorption capacities, respectively, and *K*_L_ (L mg^−1^) is the Langmuir constant.^15,40^ The dimensionless separation factor *R*_L_, used to evaluate adsorption favorability, is defined as:4
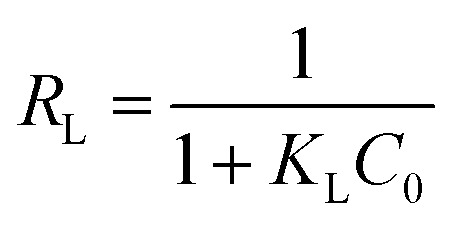
with (*R*_L_ >1) indicating unfavorable adsorption, (*R*_L_ = 1) linear adsorption, (*R*_L_ = 0) irreversible adsorption, and (0 < *R*_L_ < 1) favorable adsorption. Lower *R*_L_ values denote highly favorable adsorption.

The Freundlich isotherm (Freundlich, 1907) is expressed as:

5 *q*_e_ = *K*_f_*C*_e_^1/*n*^

where *K*_f_ (mg g^−1^ (L mg^−1^) 1/*n*) represents the adsorption capacity and 1/*n* describes adsorption intensity and surface heterogeneity. Adsorption is considered unfavorable when (1/*n* >1), homogeneous when (1/*n* = 1), and favorable when (0 < 1/*n* < 1).

The Temkin isotherm, which accounts for adsorbate–adsorbent interactions, is represented in its non-linear form as:6
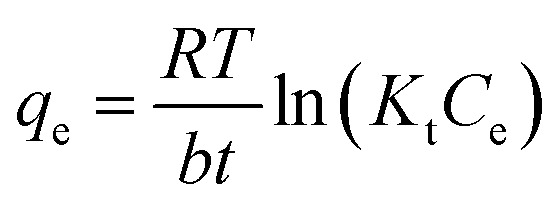
where *K*_*t*_ (L mg^−1^) is the Temkin constant, bt (J mol^−1^) is related to the heat of adsorption, *R* is the universal gas constant (8.314 J K^−1^ mol^−1^), and *T* is the absolute temperature (*K*).

The Langmuir–Freundlich isotherm was introduced to describe adsorption on heterogeneous surfaces.^41^ This model illustrates the distribution of adsorption sites on the adsorbent surface.^42^ At low adsorbate concentrations, it reduces to the Freundlich isotherm, whereas at high concentrations it approaches the Langmuir isotherm.^41^ The non-linear form of the Langmuir–Freundlich equation is expressed as:7
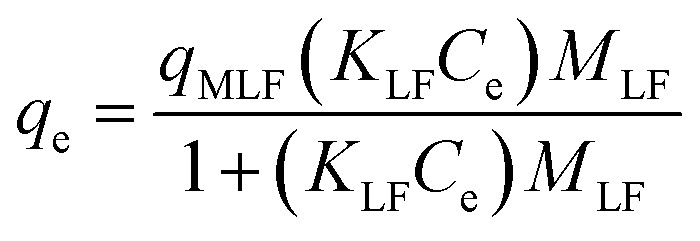
where *q*_MLF_ (mg g^−1^) represents the maximum adsorption capacity according to the Langmuir–Freundlich model, *K*_LF_ is the equilibrium constant related to the heterogeneous surface, *M*_LF_ (0–1) is the heterogeneity parameter, and *C*_e_ (mg L^−1^) is the equilibrium concentration Table 1.

**Table 1:** Isotherm parameters for RhB dye adsorption onto oak pericarp biosorbent

Models	Isotherm parameters			
Langmuir model	*Q*_max_	*K*_L_	*R* ^2^	*R*_L_
	160.809 ± 13.852	0.167 ± 0.034	0.988	0.0473
Freundlich model	*K*_f_	1/*n*	*R* ^2^	
	30.101 ± 2.195	0.510 ± 0.122	0.993	
Temkin model	*K*_*t*_	*B*_*t*_	*R* ^2^	
	2.786 ± 0.071	1.254 ± 0.011	0.925	
Langmuir–Freundlich model	*q*_MLF_	*K*_LF_	*R* ^2^	
	27.006 ± 8.427 × 10^−16^	0.167 ± 5.265 × 10^−18^	1.000	

**Fig. 13:**
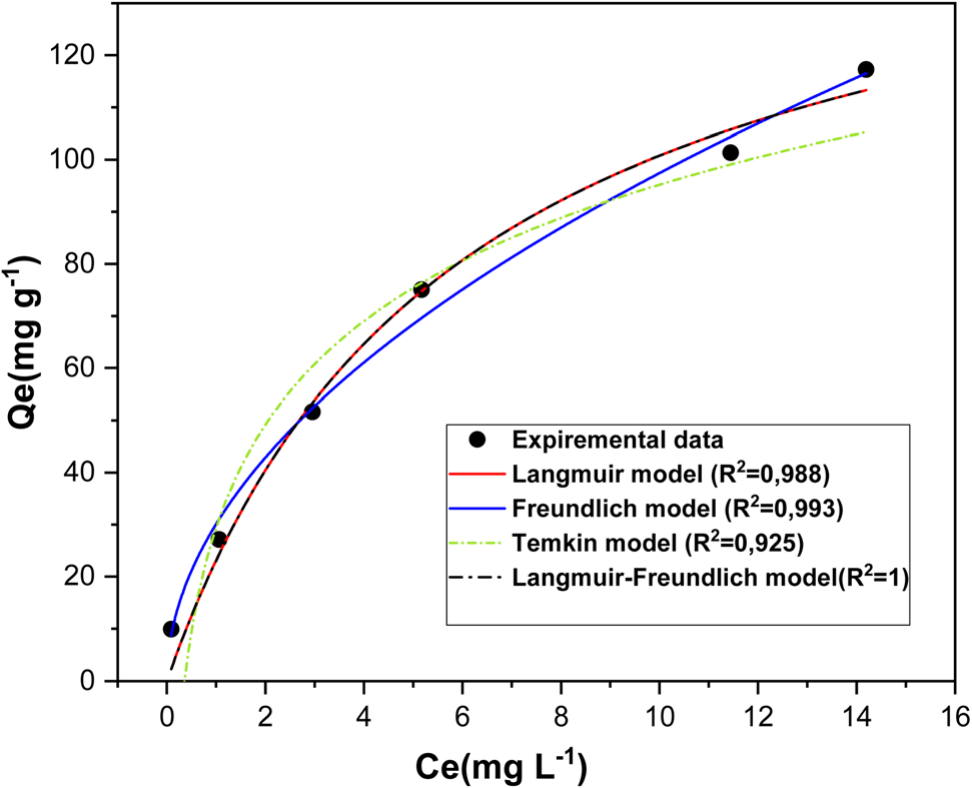
Adsorption isotherms of RhB dye on oak pericarp biosorbent at 20 °C, fitted with the Langmuir, Freundlich, Temkin and Langmuir–Freundlich models.

The fitting plots of these isotherm models are shown in Fig. 13, while the calculated parameters for the Langmuir (*q*_m_, *K*_L_), Freundlich (1/*n*, *K*_f_), Temkin (*K*_*t*_, *b*_*t*_) and Langmuir–Freundlich (*q*_MLF_, *K*_LF_) models, along with their respective *R*^2^ values for RhB dye adsorption, are summarized in 15.

**Fig. 14:**
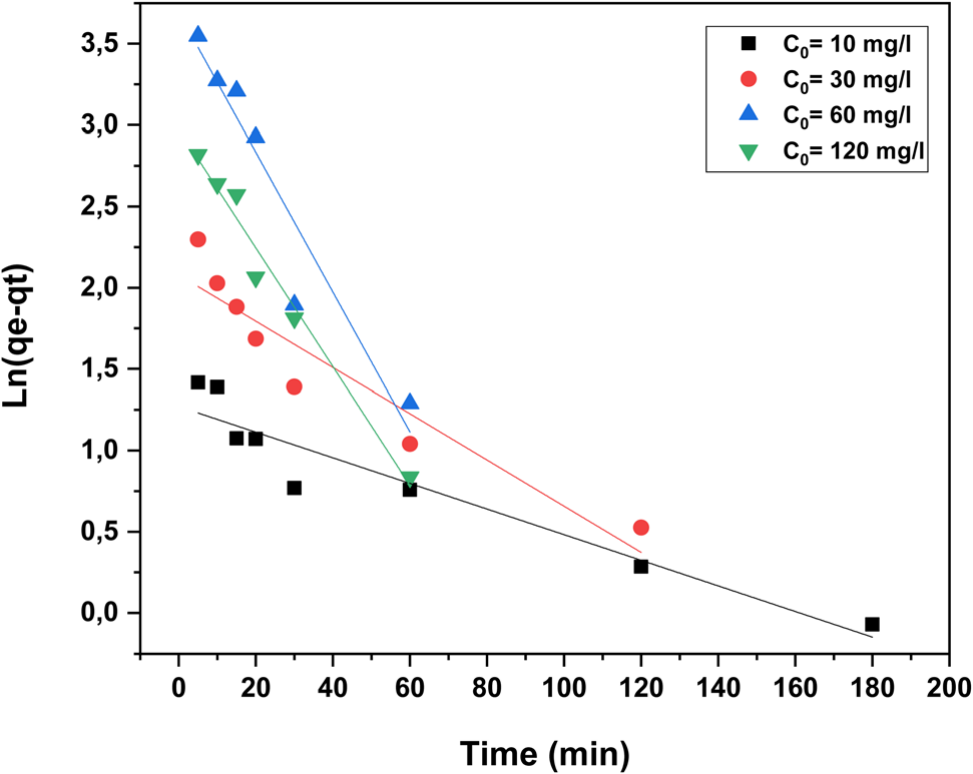
Pseudo-first-order kinetic plot for the adsorption of RhB dye onto oak pericarp biosorbent at 20 °C.

Table 1 presents the adsorption constants obtained from the Langmuir, Freundlich, Temkin, and Langmuir–Freundlich isotherms, along with their corresponding correlation coefficients (*R*^2^). The analysis indicates that the Langmuir–Freundlich model provides the best fit to the experimental data (*R*^2^ = 1.000), confirming the heterogeneous nature of the adsorbent surface and the occurrence of multilayer adsorption. The Freundlich model also demonstrates a strong correlation (*R*^2^ = 0.993), further supporting the assumption of surface heterogeneity and the presence of adsorption sites with different energies. Notably, the Freundlich constant (*n* > 1) confirms the feasibility and favorable nature of the adsorption process. The Langmuir model, with a relatively high correlation coefficient (*R*^2^ = 0.988), suggests that part of the adsorption occurs as a monolayer on homogeneous sites. In contrast, the Temkin model exhibits the lowest correlation (*R*^2^ = 0.925), implying that the assumption of a linear decrease in adsorption energy with surface coverage is less applicable in this case. Moreover, for both Langmuir and Freundlich models, the *R*_L_ and 1/*n* values fall within the range of (0–1), indicating that the adsorption of rhodamine B onto biosorbent is favorable. Overall, these findings highlight the complexity of surface interactions and confirm the predominance of heterogeneous and multilayer adsorption mechanisms (Fig. 14).

### 3.5. Adsorption kinetics study

A kinetic investigation was conducted to determine the adsorption rate constants, equilibrium adsorption capacities, and the underlying adsorption mechanisms.^36^ In this work, two kinetic models were applied to describe the adsorption behavior: the pseudo-first-order and pseudo-second-order models, at four different initial dye concentrations 10, 30, 60, and 120 mg L^−1^ for RhB while maintaining a constant temperature of 20 °C.^39^

The pseudo-first-order model proposed by Lagergren is expressed by the following equation:

8 Ln(*q*_e_ − *q*_*t*_) = ln *q*_e_ + *K*_1_*t*

where *q*_e_ and *q*_*t*_ (mg g^−1^) represent the adsorption capacities of the dye at equilibrium and at time *t*, respectively; *K*_1_ (min^−1^) is the pseudo-first-order rate constant; and *t* (min) denotes the contact time. The values of *K*_1_ and *q*_e_ were determined from the slope and intercept of the linear plot of ln(*q*_e_ − *q*_*t*_) *versus t*, for the different dye concentrations, and are summarized in Table 2.

**Table 2:** Kinetic parameters of the pseudo-first-order and pseudo-second-order models for the adsorption of RhB dye onto oak pericarp biosorbent

*C*_0_ (mg L^−1^)	*q*_e_ (exp) (mg g^−1^)	Pseudo-first-order model			Pseudo-second-order model		
		*k*_1_ (min^−1^)	*q*_e_ (cal) (mg g^−1^)	*R* ^2^	*k*_2_ (g mg^−1^ min^−1^)	*q*_e_ (cal) (mg g^−1^)	*R* ^2^
10	9.913	−0.00006575	3.562	0.915	0.0109624	9.779	0.995
30	25.661	−0.0001185	7.990	0.897	0.00481782	27.034	0.997
60	42.830	−0.00035825	40.150	0.917	0.00229635	47.573	0.994
120	95.938	−0.00030425	19.592	0.977	0.00185442	100.603	0.998

As shown in Table 2, the experimental *q*_e_ values deviate significantly from the calculated ones, suggesting that the pseudo-first-order kinetic model does not adequately describe the adsorption of RhB dye onto oak pericarp biosorbent.^36^

**Fig. 15:**
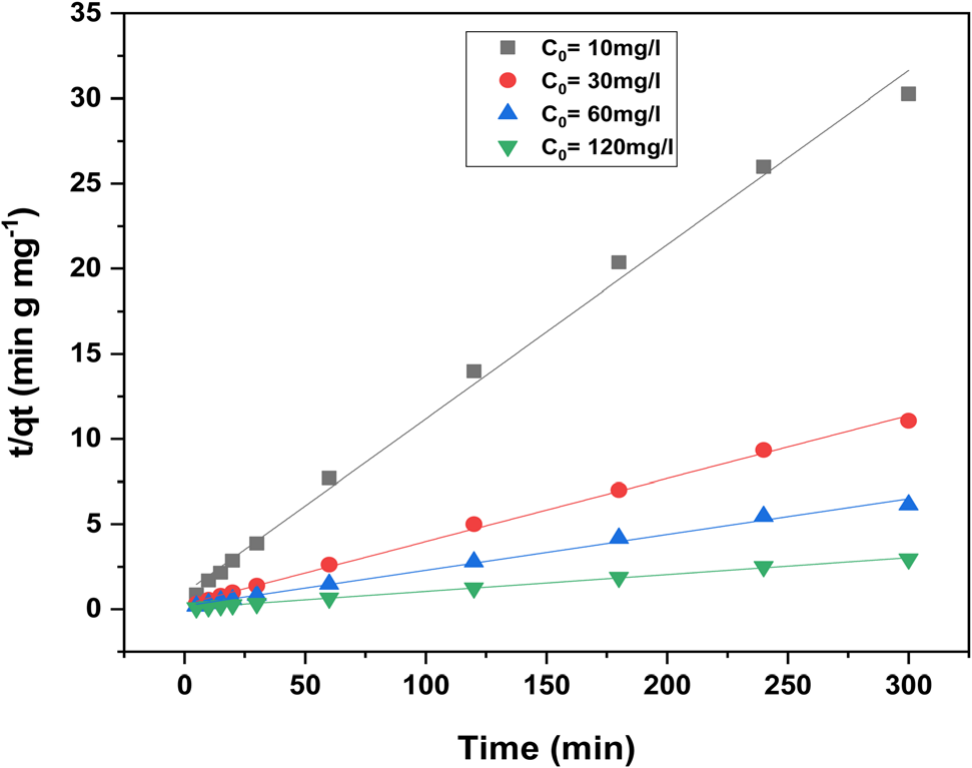
Pseudo-second-order kinetic plot for the adsorption of RhB dye onto oak pericarp biosorbent at 20 °C.

The pseudo-second-order kinetic model can be expressed as:9
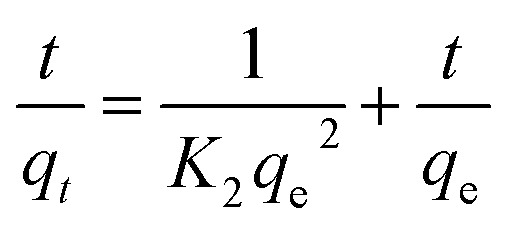
where *K*_2_ (g mg^−1^ min^−1^) represents the rate constant of the pseudo-second-order model. The kinetic parameters *q*_e_ and *K*_2_ were determined from the slope and intercept of the linear plot of *t*/*q*_*t*_*versus t*, as illustrated in Fig. 15.

Table 2 summarizes the kinetic parameters derived from the pseudo-second-order model at different initial concentrations. The experimental data showed an excellent fit to this model, as evidenced by the high correlation coefficient (*R*^2^ > 0.998) obtained for RhB dye. Moreover, the adsorption capacity predicted by the model (*q*_e_, cal) was in close agreement with the experimentally measured values (*q*_e_, exp) across the four concentrations tested. These findings confirm that the pseudo-second-order model provides a reliable description of the adsorption kinetics of RhB dye onto oak pericarp biosorbent.

### 3.6. Thermodynamic studies

Thermodynamic analysis is a key approach to assess the temperature dependence of the adsorption equilibrium constant.^22^ In this work, the effect of temperature on the removal efficiency was investigated under optimum conditions at four different temperatures (293, 303, 313 and 323 K) to evaluate the thermodynamic behavior of the adsorption process.^43^

The main thermodynamic parameters, namely the standard Gibbs free energy change (Δ*G*°), the standard enthalpy change (Δ*H*°), and the standard entropy change (Δ*S*°), provide valuable insights into the feasibility, spontaneity, and nature of adsorption.^44^

The standard Gibbs free energy change (Δ*G*°, kJ mol^−1^) was determined according to eqn (10):

10 Δ*G*° = −*RT*ln *K*_c_

where *R* is the universal gas constant (8.314 J mol^−1^ K^−1^), *T* the absolute temperature (*K*), and *K*_c_ the thermodynamic equilibrium constant. The latter was calculated using eqn (11):11
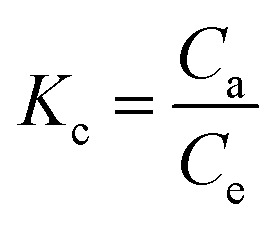
with *C*_a_ (mg L^−1^) and *C*_e_ (mg L^−1^) representing the equilibrium dye concentrations on the adsorbent and in the solution, respectively.

The interrelationship between Δ*G*°, Δ*H*°, and Δ*S*° is expressed as:

12 Δ*G*° = Δ*H*° − *T*Δ*S*°

**Fig. 16:**
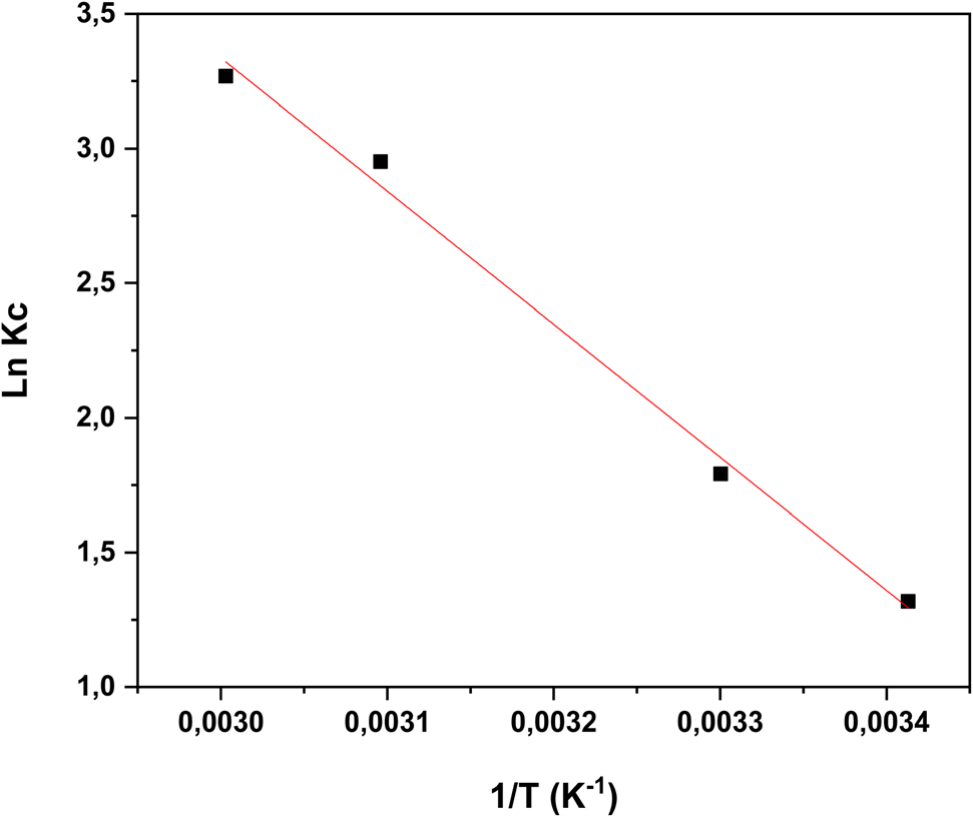
Van't Hoff plots for the adsorption of RhB dye onto oak pericarp biosorbent.

13
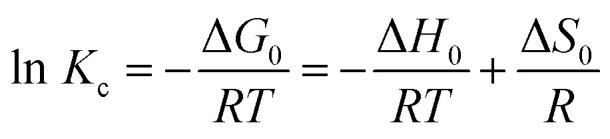
where Δ*H*° (kJ mol^−1^) and Δ*S*° (kJ mol^−1^ K^−1^) were determined from the slope and intercept of the van't Hoff plot of ln*K*_*c*_*versus* 1/*T* (Fig. 16).

Table 3 summarizes the thermodynamic parameters determined in this work. The negative Δ*G*° values indicate that the adsorption of dye onto the biosorbent occurs spontaneously and is energetically favorable. The positive value of Δ*H*° demonstrates that the process is endothermic, and its relatively high magnitude suggests that the adsorption occurs mainly through chemisorption. Furthermore, the positive Δ*S*° values indicate an increase in entropy at the solid–solution interface with rising temperature, thereby supporting the enhanced feasibility of adsorption at elevated temperatures.^45^

**Table 3:** Thermodynamic parameters of RhB dye adsorption on the oak pericarp biosorbent

Temperature (K)	Thermodynamic parameters			
	Δ*G*° (kJ mol^−1^)	Δ*H*° (kJ mol^−1^)	Δ*S*° (kJ mol^−1^ K^−1^)	*R* ^2^
293	−0.511			
303	−0.972			
313	−2.726	41.087	150.994	0.994
323	−3.448			

### 3.7. Reusability experiments

**Fig. 17:**
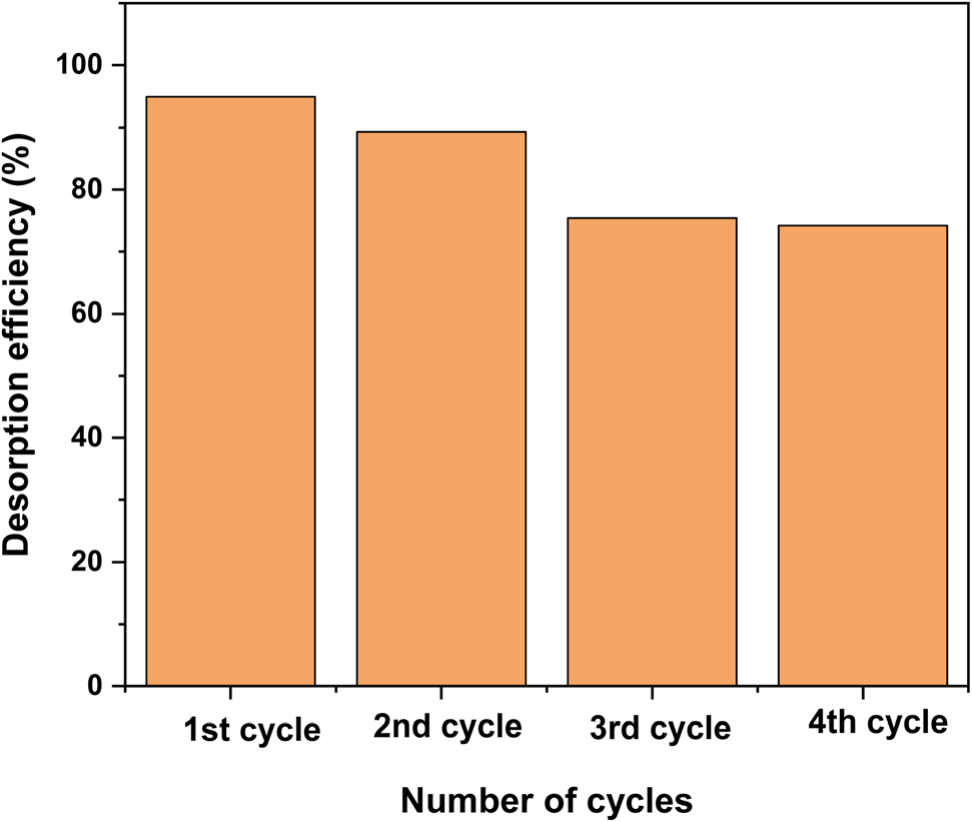
Desorption efficiency of RhB dye using oak pericarp biosorbent.

The reusability of a biosorbent is a key factor for practical applications, as an efficient material should combine high adsorption capacity with good regeneration performance. Fig. 17 illustrates the adsorption capacity of the regenerated biosorbent over successive cycles. As observed, the adsorption efficiency gradually decreased with repeated use. After the fourth cycle, the desorption efficiency dropped from 94.92% to 74.19% for RhB dye compared with the first cycle.^46^ This reduction can be attributed to the progressive loss or deactivation of surface functional groups during each regeneration step.

Despite this decrease, the biosorbent demonstrated easy recovery and relatively high regeneration efficiency, confirming its potential for repeated use. These findings suggest that the biosorbent could be considered an economically viable option for real wastewater treatment applications. Moreover, the spent biosorbent was collected for further investigation in future research.^22^

Overall, the promising reusability and regeneration performance of the biosorbent not only enhance its economic feasibility but also contribute to sustainable environmental management. By minimizing sorbent consumption and reducing secondary waste generation, the biosorbent represents a potential eco-friendly solution for large-scale wastewater treatment.

### 3.8. Adsorption mechanism

The adsorption mechanism of rhodamine B (RhB) onto the oak acorn pericarp biosorbent can be explained by a combination of physicochemical interactions. The FTIR spectra revealed the involvement of hydroxyl (–OH), carbonyl (C=O), and aromatic groups, suggesting that hydrogen bonding and π–π interactions play a significant role in dye adsorption. In addition, at acidic pH values (below the pH_pzc_), the biosorbent surface becomes positively charged, favoring electrostatic attraction between the cationic RhB molecules and negatively charged sites formed through functional group dissociation. The heterogeneous porous structure observed by SEM further enhances dye diffusion and surface accessibility. Overall, the adsorption process is controlled by multiple mechanisms, including electrostatic attraction, hydrogen bonding, and π–π stacking interactions between the aromatic structures of RhB and lignin components of the biosorbent, leading to efficient and stable dye removal.

### 3.9. Comparison with reported biosorbents

Table 4 presents a comparative analysis of the adsorption performance of the oak acorn pericarp biosorbent with other plant-based materials previously reported for rhodamine B (RhB) removal. The maximum adsorption capacity (*q*_max_) obtained in this study was relatively higher than several reported biosorbents, such as banana peel, rice husk, Kappaphycus alvarezii, Gracilaria salicornia and Gracilaria edulis, demonstrating the superior efficiency of the developed material. This enhanced performance can be attributed to the rich surface chemistry and porous structure of the oak pericarp, which provide numerous active sites for dye binding. Moreover, the presence of mesopores facilitates rapid diffusion of RhB molecules toward the internal adsorption sites, contributing to higher adsorption capacity and faster equilibrium attainment. Overall, these results highlight the potential of oak acorn pericarp as an efficient, sustainable, and low-cost biosorbent for dye-contaminated wastewater treatment.

**Table 4:** Comparison of adsorption performance of the oak acorn pericarp biosorbent for RhB dye with other reported adsorbents

Adsorbent	*q*_max_ (mg g^−1^)	References
Oak acorn pericarp biosorbent	160.809	This work
Banana peel	81.07	12
Kappaphycus alvarezii (KA)	9.84	13
Gracilaria salicornia (GS)	11.03	13
Gracilaria edulis (GE)	8.96	13
Raw rice husk biochar	40	14

## 4. Conclusion

This study demonstrated the successful preparation and characterization of a novel biosorbent derived from oak acorn pericarp for the removal of rhodamine B from aqueous solutions. Structural and surface analyses (FTIR, SEM-EDX, BET, TGA, XRD, and pH_pzc_) confirmed the presence of functional groups and porous structures that facilitated the adsorption process. Batch experiments revealed that adsorption performance was strongly influenced by pH, contact time, and dye concentration, with optimal removal achieved under acidic conditions.

The adsorption behavior was best described by the Langmuir–Freundlich isotherm, indicating heterogeneous multilayer adsorption, while kinetic modeling highlighted the predominance of a pseudo-second-order mechanism, suggesting chemisorption. Thermodynamic evaluations confirmed that the process was feasible, spontaneous, and endothermic. Importantly, reusability tests demonstrated that the oak pericarp biosorbent maintained substantial adsorption capacity over several regeneration cycles.

The conclusion was rewritten to be more concise, summarizing the main quantitative results, such as the maximum adsorption capacity (160.809 mg g^−1^), pseudo-second-order kinetics, and Langmuir–Freundlich model fitting.

Overall, the findings highlight oak acorn pericarp as a low-cost, eco-friendly, and sustainable biosorbent with strong potential for wastewater treatment applications. Future work could focus on scaling up the process, testing performance with real industrial effluents, and exploring surface modifications to further enhance adsorption capacity and selectivity.

## Conflicts of interest

The author declare that there are no conflicts of interest.

## Supplementary Material

RA-015-D5RA07130E-s001

## Data Availability

All data supporting the findings of this study are included within the manuscript. No additional datasets or supporting files are available.
